# Micro-Tensile Behavior of Mg-Al-Zn Alloy Processed by Equal Channel Angular Pressing (ECAP)

**DOI:** 10.3390/ma11091644

**Published:** 2018-09-07

**Authors:** Kristián Máthis, Michal Köver, Jitka Stráská, Zuzanka Trojanová, Ján Džugan, Kristýna Halmešová

**Affiliations:** 1 Department of Physics of Materials, Faculty of Mathematics and Physics, Charles University, Ke Karlovu 5, 121 16 Praha 2, Czech Republic; mathis@met.mff.cuni.cz (K.M.); jitka.straska@mff.cuni.cz (J.S.); 2 Nuclear Physics Institute of the CAS, 250 68 Řež, Czech Republic; 3 COMTES FHT, Průmyslová 995, 334 41 Dobřany, Czech Republic; kover_m@centrum.sk (M.K.); jdzugan@comtesfht.cz (J.D.); kristyna.halmesova@comtesfht.cz (K.H.)

**Keywords:** magnesium alloy, equal channel angular pressing, processing route, miniaturized tensile tests, slip systems, twinning

## Abstract

Commercially available AZ31 magnesium alloy was four times extruded in an equal rectangular channel using three different routes (A, B, and C). Micro tensile deformation tests were performed at room temperature with the aim to reveal any plastic anisotropy developed during the extrusion. Samples for micro tensile experiments were cut from extruded billets in different orientations with respect to the pressing direction. Information about the microstructure of samples was obtained using the electron back-scatter diffraction (EBSD) technique. Deformation characteristics (yield stress, ultimate tensile stress and uniform elongation) exhibited significant anisotropy as a consequence of different orientations between the stress direction and texture and thus different deformation mechanisms.

## 1. Introduction

The drive for product miniaturization in various application fields, including biomedicine, the watchmaking industry and communication technologies has significantly increased the demand for metallic micro-parts. There are two approaches for their manufacturing: (i) Micro-machining (electron discharge-, laser- or focused ion beam micro-machining or etching techniques) or (ii) micro-forming when products have a high surface quality and a near-net shape can be obtained in few steps. Consequently, the advantage of micro-forming technology in comparison to micro-machining is the lower cost, given by a higher production rate [1]. In both cases, the so-called size-effect has to be taken into the account. At the microscale, materials cannot be regarded as homogeneous as the microstructural size can be similar to or larger than the parts’ dimension. This means that only a few grains are present in the cross-section of a semi-product or micro-part. This makes the downscaling of the conventional forming techniques difficult as the role of the orientation and size of every single grain substantially influences the process flow. The micro-machined products can also behave unexpectedly; deformation anisotropy, scatter in flow stress, size-dependent tribology properties or non-linear increases to the specific cutting energy have been observed [2]. 

The above-listed difficulties can be overcome by using ultra-fine-grained (UFG) materials. The submicron or nanometer grain size of UFGs, which gains a large number of grains per cross-section of a component, ensures the reproducibility of the physical properties. Further, UFG materials usually exhibit higher strength (cf. the Hall-Petch relation) and superplastic behavior at elevated temperatures [3].

Magnesium alloys are very popular in the structural applications as they are among the lightest structural materials. The application of severe plastic deformation (SPD) methods results in the formation of an ultra-fine microstructure. In the last two decades, equal channel angular pressing (ECAP) has become the most popular and the most intensively studied SPD technique for magnesium alloys. As it was shown by Kim [4] and Estrin [5], ECAP and the micro-extrusion can be integrated into a single processing flow, which results in the production of high-strength micro-parts. Despite the popularity of the ECAP process, deformation behavior on the micro-scale is rarely studied. There are only a few papers (Al [6,7], Mg [8], Ti [9]) that deal with this topic. 

In this work, the mechanical properties of ECAP processed AZ31 magnesium alloy was studied using micro tensile tests (M-TT) [10]. The deformation behavior as a function of the processing route and specimen orientation with respect to the pressing direction is discussed in detail. The EBSD data were used for the interpretation of the achieved results.

## 2. Experimental Section

**Figure 1:**
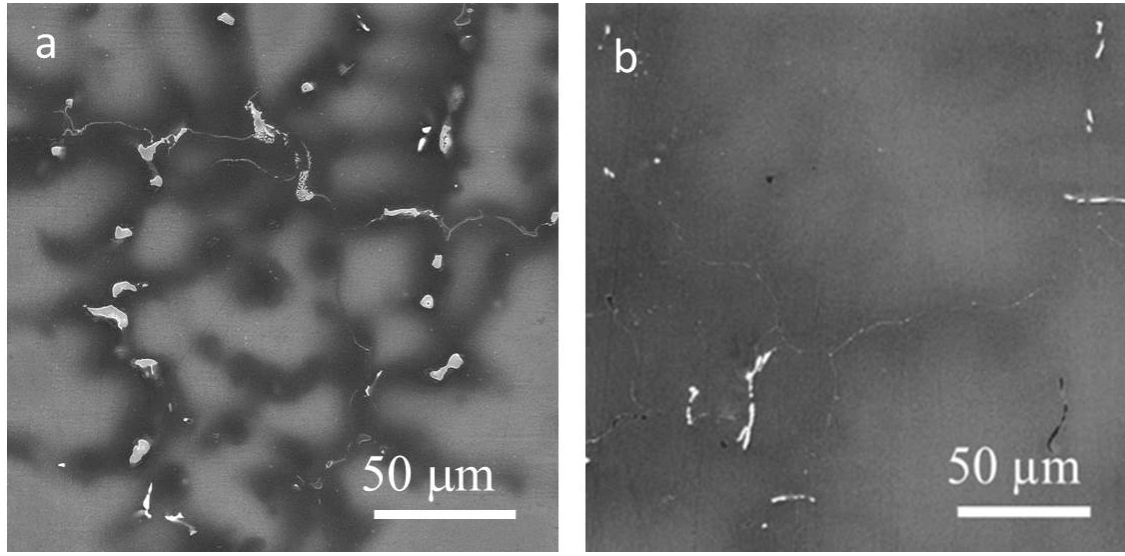
SEM micrographs of the cast alloy before annealing (**a**) and after annealing (**b**).

Sand cast AZ31 magnesium alloy with a nominal composition of Mg—3 wt% Al—1 wt% Zn was investigated in this study. The material was subjected to standardization annealing for 18 h at 390 °C. The microstructure of the alloy before and after annealing is reported in Figure 1a,b. Small precipitates. visible in Figure 1a, are the Mg_17_Al_12_ electron compound. After standardization annealing, these particles were mostly dissolved as is obvious from Figure 1b. The grain size of the annealed alloy was 300 μm. Samples depicted hereafter “as cast” are after standardization annealing.

**Figure 2:**
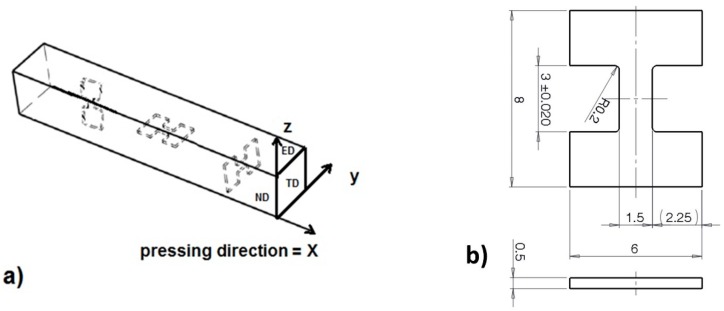
(**a**) The sample coordinate system and the orientation of the specimens. (**b**) Dimensions of the specimens used for micro-tensile tests.

The ECAP was performed on 10 × 10 × 100 mm^3^ billets at a temperature of 250 °C and up to 4 passes, following routes A, B_c_ and C [3]. All samples were pressed at a speed of 10 mm/min through a die consisting of rectangular channels (inner angle ϕ = 90°, outer curvature ψ = 0°) with the same cross-section of 10 × 10 mm^2^. In order to study the orientation dependence of micro-tensile properties, samples with their longitudinal axis parallel to the ED-, TD- and ND-planes, this was parallel to the extrusion (pressing), transversal- and normal-directions, respectively, were machined from the billets (Figure 2a). The sample shape (*l* = 3 mm, *w* = 1.5 mm, *t* = 0.5 mm) is depicted in Figure 2b. 

The tensile tests were performed at room temperature with a strain rate of 10^−3^ s^−1^ in a servo-electric test machine M-TT (Material testing Technology. Wheeling, IL, USA) with a 5 kN load capacity. The strain was measured using the Digital Image Correlation (DIC) technique (Dantec Dynamics AVS, Skovlunde, Denmark), implemented in Aramis software. The sample coordinate system used for texture representation is depicted in Figure 2a. The samples were grinded by SiC papers using diamond suspensions down to 0.25 μm. A final surface treatment for EBSD was performed by ion polishing using a Gatan Precision Ion Polishing System (PIPS ion mill, (Gatan, Pleasanton, CA, USA)) at 4 kV and an incidence angle of 6°. The examination of microstructures and textures was carried out using a Quanta field emission gun (FEG) scanning electron microscope (Thermo Fisher Scientific, Waltham, MA, USA) operated at 10 kV equipped with the electron back-scattered diffraction (EBSD) camera. Areas of approximately 250 × 250 μm^2^ with a step size of 0.2 µm were examined. The average grain size was estimated form the EBSD data. 

## 3. Results and Discussion

### 3.1. Initial Microstructure and Texture

**Figure 3:**
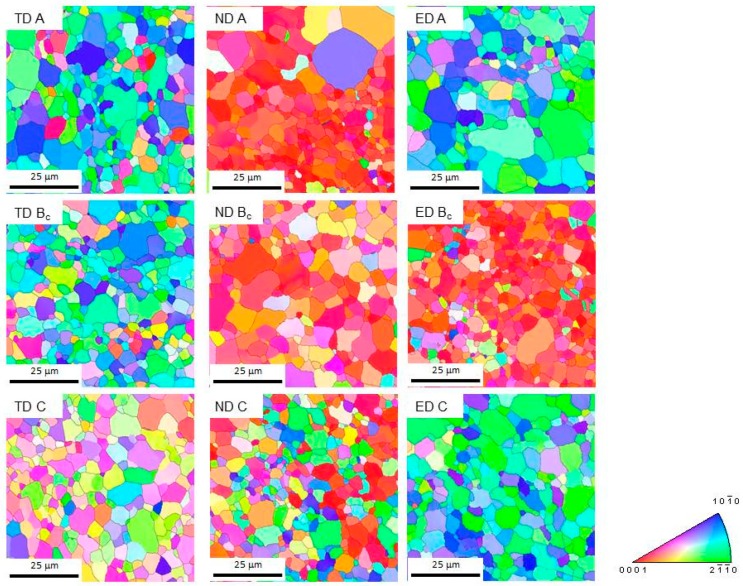
Inverse pole figure maps obtained for the particular planes and ECAP routes.

The inverse pole figure maps of the initial microstructures after four passes are shown in Figure 3. Areas smaller (80 µm × 80 µm) than the scanned one are presented in order to see the details. It is obvious that the ECAP process resulted in a substantial refinement of the microstructure—small grains were formed on all planes for every processing route. The average grain sizes and the fraction of high angle grain boundaries (HAGB, >15°) are listed in Table 1.

**Figure 4:**
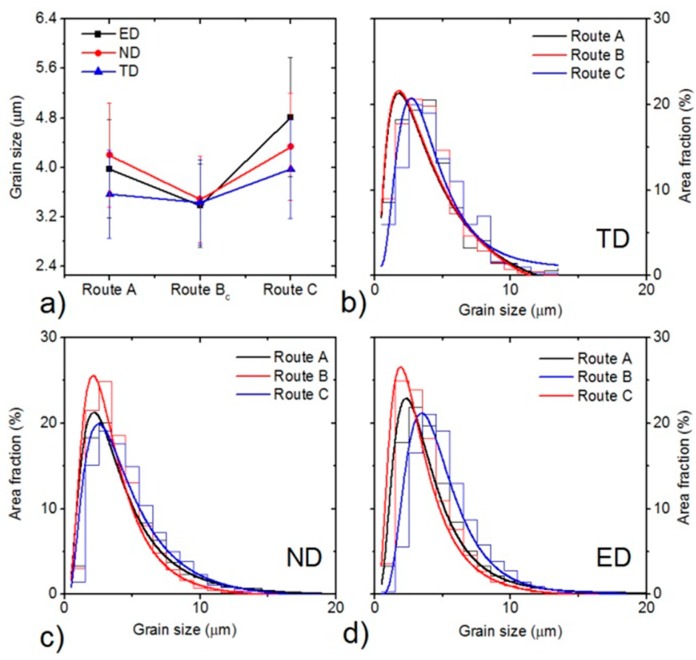
Average grain sizes of the particular samples (**a**). Grain size distributions for the particular ECAP routes in the transversal plane (**b**), normal plane (**c**) and extrusion plane (**d**).

The average grain size on all planes was almost the same for route B_c_. In contrast, the grain refinement was orientation dependent for the two other processing routes. For both routes A and C, it is obvious (Figure 3) that the microstructures have a significant character of bimodality—the area fractions of larger grains exceed that for route B_c_. This effect can be characterized by a log-normal distribution of the grain sizes (only grains with misorientation angles larger than 15° were considered). The narrowest distribution in all examined directions was found for route B_c_ (Figure 4), whereas the widest for route C. Further, the fraction of HAGBs was also the largest for route B_c_. This result is in good agreement with many previous works, which have reported that route B_c_ leads to the most rapid evolution of ultrafine microstructure [3,11].

**Figure 5:**
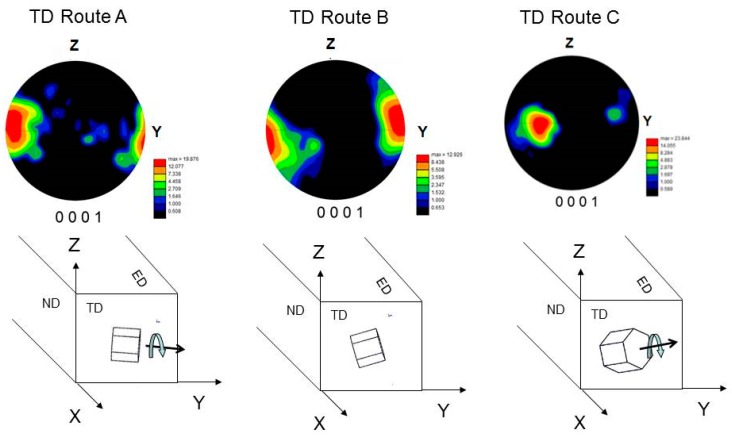
(0001) EBSD pole figures for the TD plane and the corresponding schemes of the prevailing grain orientations.

During the ECAP process, simple shear was the dominant deformation mode, which is accompanied with large crystal rotations [12]. Their magnitude depended on the orientation of the active slip system with respect to the shear stress. The maximum value was reached when the Burgers vector of active dislocations was perpendicular to the shear direction. The grains tended to reach a stable orientation as the strain increased. This can be simply fulfilled for route C as between two consecutive passes only the shear strain sign was changed, which did not cause further rotations. However, this was not the case for route A nor for route B_c_, where every orientation reached in the *n*th pass was unstable with respect to shear in the (*n +* 1)th pass [12]. This led to activation of various slip systems, particularly the non-basal ones, as was experimentally proved in our previous work [11]. The non-basal dislocations tended to form sessile configurations, which were the seeds for new grain nuclei. This effect was most pronounced at the grain boundaries, where the stress concentrations were the largest. Therefore, the larger grains were surrounded with a chain of smaller grains, according to the observations of Figueiredo [13]. The (0001) pole figures showing the texture of the TD plane (this is perpendicular to the extrusion direction), are shown in Figure 5. Following the notation of Beausir [14] and Krajňák [15], two texture components A (route A and B_c_) and B (route C) were observed. As it is discussed in detail in these papers, both texture components form after the first ECAP pass. The type A texture component was a result of extension twinning activated during the compression of the billet in the feed-in (vertical) channel and the second-order pyramidal (<*c+a*>) slip. The type B texture appeared as a consequence of the increased activity of the basal slip. As the number of passes increased, the different routes developed different textures owing to the mutual orientation of the shear planes of the consecutive passes. For route A, when no sample rotation took place, the repeated pressing always caused extension twinning and a <*c+a*>-slip in the vertical channel. This led to the strengthening of the A-type texture component. For route B_c_, the increased activity of a <*c+a*>-slip has been observed [11], which led to the preservation of the A-type texture component. However, some B-type components also remained. For route C, after the first pass, the grains rotated into an orientation favorable for the basal slip. As it is discussed above, in this case only the shear sign, not the direction changed. Consequently, the B-type texture component became dominant. The prevailing orientations of grains within the billet are exemplified by the hexagonal prisms in Figure 5.

### 3.2. Mechanical Properties

**Figure 6:**
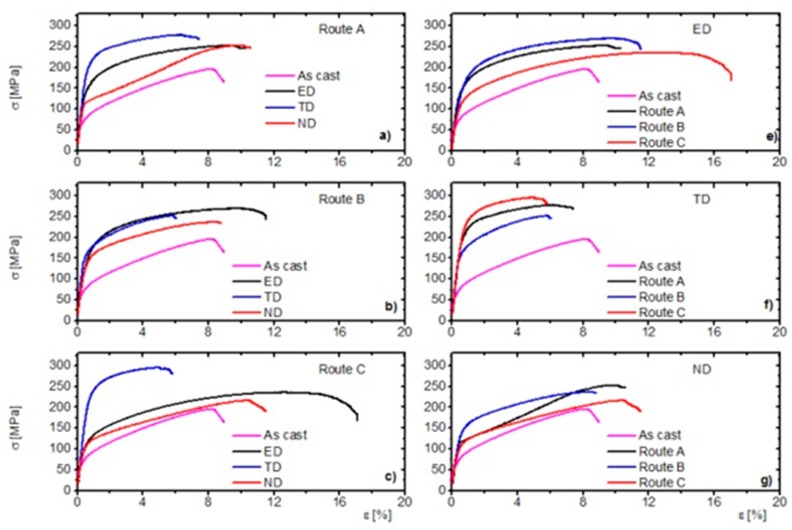
Stress-strain curves (**a**–**c**) for particular routes as a function of the tensile direction and (**e**–**g**) for the particular directions as a function of ECAP routes.

The stress-strain curves for the particular routes and directions are plotted in Figure 6. The characteristic parameters yield stress (YS), ultimate tensile strength (UTS) and uniform elongation are listed in Table 2. All tests were repeated twice and the scatter between the values was below 2%. It is obvious that the ECAP process increased the yield stress and tensile strength in comparison to the initial state.

The main contribution to this increment was given by the refinement of the microstructure according to the Hall-Petch rule [16,17]. The results of the micro tensile tests on the ECAPed samples clearly showed that both the processing routes and the direction of the tensile tests had a substantial impact on the mechanical properties. Since the grain sizes were similar, the texture was the foremost parameter responsible for the variance of the stress values [18]. The texture determined the Schmid factor (SF) of the particular deformation mechanism. Based on the EBSD measurement, the SFs for uniaxial tension, parallel to the particular direction, were calculated by TSL OIM Analysis 7 software for $\left(0001\right)\;\langle 11\bar{2}0\rangle$ basal, $\left\{10\bar{1}0\right\}\;\langle 11\bar{2}0\rangle$ prismatic, $\left\{101\bar{2}\right\}\;\langle 11\bar{23}\rangle$ 2nd order pyramidal and $\left\{10\bar{1}2\right\}\;\langle 10\bar{1}1\rangle \;$ extension twinning systems (Table 3).

It was obvious that the SF for the pyramidal <*c+a*>-slip was almost the same for all routes and directions. The activation of twinning was generally easier in ND, and for ED after route B processing. The SF for the prismatic <*a*>-slips also had high values, except in ED for routes A and C or in TD for route B, respectively. There was a large scatter in the SF values for the basal <*a*>-slip. The activation of this mechanism was facilitated rather in ED. In the following two sections we discuss separately the influence of the ECAP routes and tensile directions on the mechanical properties in terms of the determined SFs.

#### 3.2.1. Influence of the Tensile Direction on Mechanical Properties for the Particular ECAP Routes

Generally, it can be concluded that the highest YS and the lowest ultimate elongation were observed in TD for all routes. In contrast, the lowest YS value was observed for ND. The uniform elongations had similar values for ED and ND. The ultimate tensile strength was also the lowest for ND, except route A, where its value was a bit higher than that for ED.

Route A (Figure 6a)—the SF_basal_ and SF_twinning_ values were the lowest in the TD direction. The deformation was realized mainly by the prismatic <*a*>-slip and the pyramidal <*c+a*>-slip. The critical resolved shear stress for these mechanisms at room temperature was several orders higher compared to those for the basal slip and extension twinning [19]. In ND, the deformation curve had a characteristic S-shape, which was clear evidence of the activation of extension twinning [20]. One can argue that in fine-grained structures the probability of twinning is low [21]. However, we have to keep in mind that the structure was bimodal—larger grains (*d >* 10 µm) were also present. In these grains, the extension twins were easily nucleated [22]. As a consequence of the rapid twin growth, the ultimate elongation exceeded that for TD. The UTS value was similar to that for ED owing to the secondary hardening when the twin growth terminates [20]. In ED there was the highest SF_basal_ value but the SF_twinning_ value was zero. Consequently, the activation of the <*c+a*>-slip was required for plastic deformation, according to the von Mises rule [23]. The YS was between the ED and ND owing to the interaction of basal and non-basal dislocations. The uniform elongation was quite large owing to the activity of the basal slip [15].

Route B (Figure 6b)—in the ND direction, the ratio of the SF values was similar to that of route A, thus YS and UTS were the lowest. The activity of twinning was lower than that of the previous case (cf. the shape of the curves), which was most probably given by a more uniform grain size. Despite the highest SF_basal_ and lowest SF_prismatic_ values, the YS slightly exceeded of that for ED, where the ratio of these SFs was exactly the opposite. The reason can be given in the fact that the higher SF_twinning_ in ED lowers the YS and increases the ultimate elongation. 

Route C (Figure 6c)—in this case, this situation is almost analogical to route A. The highest YS and UTS and the lowest ultimate elongation were observed for TD owing to the low SF_basal_ and high SF_prismatic_ and SF_pyramidal_ values. The ND again had the lowest strength value and the curves indicated (but not as evidently as for route A) activation of twinning. In ED, the basal slip and the <*c+a*>-dislocation activity was expected, whereas the probability of twinning was close to zero. Thus, the ultimate elongation was the largest for ED. 

#### 3.2.2. Influence of the ECAP Routes on the Mechanical Properties in the Particular Tensile Directions

**Figure 7:**
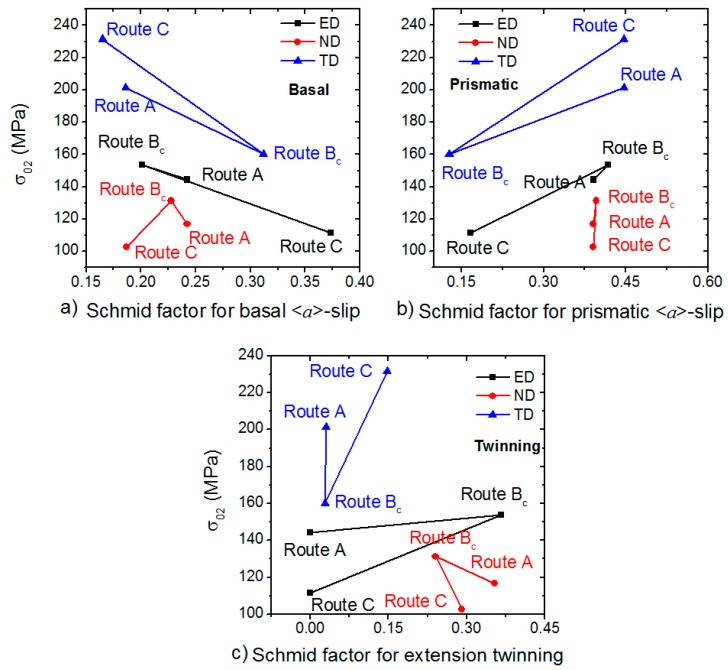
Dependence of the yield stress on the Schmid factor for (**a**) the basal <*a*>-slip, (**b**) the prismatic <*a*>-slip; and(**c**) extension twinning.

**Figure 8:**
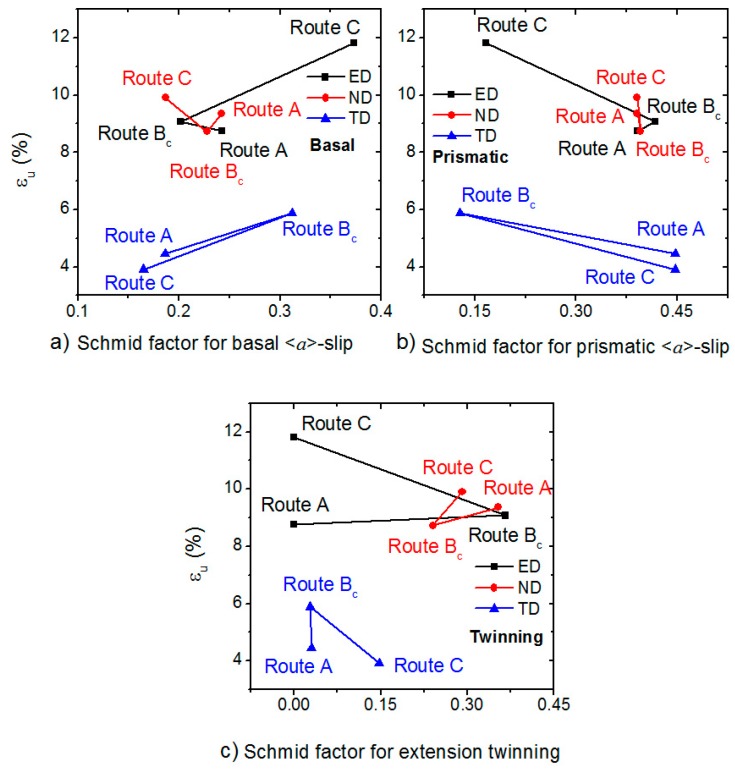
Dependence of the uniform elongation on the Schmid factor for (**a**) the basal <*a*>-slip, (**b**) the prismatic <*a*>-slip and (**c**) extension twinning.

In order to facilitate the overview of the SFs and the mechanical parameters relations, we plotted the SF dependence of YS and the ultimate elongation in Figure 7 and Figure 8.

ED (Figure 6e, Figure 7 and Figure 8a)—the YS increases with the decreasing SF_basal_ and increasing SF_prismatic_. The higher critical resolved shear stress (CRSS) of the prismatic <*a*>-slip was in the background of this effect. Further, the prismatic <*a*> dislocations acted as forest dislocations for the movement of the basal <*a*> ones [24], which caused hardening. The high SF_twinning_ value was responsible for softening in the route B_c_ case [25]. The uniform elongation behaved in the opposite way and increased with increasing SF_basal_ and decreased with decreasing prismatic SF_prismatic_. It seems that the twinning was responsible for the similar values of route A and B_c_ elongations.

TD (Figure 6f, Figure 7, and Figure 8b)—in TD, the values were the opposite compared to that for ED. The strength values for route C exceeded that for the other two routes. The uniform elongations were almost the same for all routes. The reason for this behavior is given by the fact that the SF_basal_ value was the largest and the SF_prismatic_ value was the smallest for route B_c_, respectively. Accordingly, the SF dependence of the uniform elongation was the same as for ED. 

ND (Figure 6g, Figure 7, and Figure 8c)—the YS values were similar to ED (YS_B_ > YS_A_ > YS_C_). The UTS was the highest for the route A. The ultimate elongation values are in order route C > route A > route B. In this direction, the SF values were close to each other, which caused the mechanical parameters to be similar.

## 4. Conclusions

The micro tensile properties of 4x ECAPed AZ31 magnesium alloy were examined as a function of the ECAP processing route and tensile direction. The variation of the mechanical properties for the particular samples was substantiated by the different textures, causing various conditions for the deformation mechanisms. The processing routes and the direction of the tensile tests had a substantial impact on the mechanical properties. The following particular conclusions can be drawn.

Influence of the Processing Routes:
  - The Schmid factors for the basal <*a*> slip and extension twinning were foremost responsible for the values of uniform elongation.
  - The highest values of yield stress and yield strength were found for samples with the highest Schmid factor for the prismatic <*a*> and pyramidal <*c+a*>-slips and the lowest Schmid factor for basal <*a*> dislocations.
Influence of the Tensile Direction:
  - In ED and TD, the yield stress was determined with the ratio of Schmid factors for basal <*a*> and prismatic <*a*> slips. The higher the value, the lower the yield stress. In ND, the twinning activity was significant for all routes.

## Figures and Tables

**Table 1:** Average grain sizes and fractions of high angle grain boundaries for particular ECAP routes and planes, as estimated from EBSD measurements.

Route/Plane	TD		ND		ED	
ECAP Route	Grain Size (µm)	Fraction of HAGBs	Grain Size (µm)	Fraction of HAGBs	Grain Size (µm)	Fraction of HAGBs
A	3.57	85.5%	4.20	89.7%	3.98	91.0%
B_C_	3.43	92.1%	3.48	93.8%	3.37	90.2%
C	3.97	86.9%	4.33	89.5%	4.81	89.6%

**Table 2:** Mechanical characteristics as a function of the processing route and direction of the tensile testing.

Route	Plane	Yield Stress (σ_02_)	Ultimate Tensile Strength (σ_max_)	Uniform Elongation (ε_u_)
		[MPa]	[MPa]	[%]
Route A	TD	201	275	4.4
	ND	117	264	9.4
	ED	144	253	8.8
Route B_c_	TD	160	261	5.9
	ND	131	242	8.7
Route C	TD	231	295	3.9
	ND	103	216	9.9
	ED	112	236	11.8
Initial	-	57	195	8.1

**Table 3:** Schmid factors for the particular deformation mechanisms in particular billet planes calculated for uniaxial tension.

Plane_Route	Basal Slip	Prismatic Slip	Pyramidal <*c*+*a*> Slip	Extension Twinning
TD_A	0.19	0.45	0.44	0.03
TD_B	0.31	0.13	0.43	0.02
TD_C	0.17	0.45	0.43	0.15
ND_A	0.24	0.39	0.42	0.35
ND_B	0.23	0.40	0.41	0.24
ND_C	0.19	0.45	0.44	0.29
ED_A	0.32	0.17	0.43	0
ED_B	0.20	0.42	0.43	0.36
ED_C	0.37	0.22	0.38	0
